# Isolation and characterization of a new *Methanoculleus bourgensis* strain KOR-2 from the rumen of Holstein steers

**DOI:** 10.5713/ajas.18.0409

**Published:** 2018-07-26

**Authors:** Urantulkhuur Battumur, Manhee Lee, Gui Sek Bae, Chang-Hyun Kim

**Affiliations:** 1 Graduate School of Future Convergence, Hankyong National University, Anseong 17579, Korea; 2 School of Animal Science and Biotechnology, Mongolian University of Life Sciences, Ulaanbaatar 17024, Mongolia; 3 Department of Animal Life and Environment Science, General Graduate School, Hankyong National University, Anseong 17579, Korea; 4 Department of Animal Science and Technology, Chung-Ang University, Anseong 17546, Korea; 5 Department of Animal Life and Environment Science, Hankyong National University, Anseong 17579, Korea

**Keywords:** Methanogen, Rumen, 16S rRNA, *mcrA*, *Methanoculleus bourgensis*

## Abstract

**Objective:**

To isolate and identify new methanogens from the rumen of Holstein steers in Korea.

**Methods:**

Representative rumen contents were obtained from three ruminally cannulated Holstein steers (793±8 kg). Pre-reduced media were used for the growth and isolation of methanogens. Optimum growth temperature, pH, and sodium chloride (NaCl) concentration as well as substrate utilization and antibiotic tolerance were investigated to determine the physiological characteristics of the isolated strain. Furthermore, the isolate was microscopically studied for its morphology. Polymerase chain reaction of 16S rRNA and *mcrA* gene-based amplicons was used for identification.

**Results:**

One strain designated as KOR-2 was isolated and found to be a non-motile irregular coccus with a diameter of 0.2 to 0.5 μm. KOR-2 utilized H_2_+CO_2_ and formate but was unable to metabolize acetate, methanol, trimethylamine, 2-propanol, and isobutanol for growth and methane production. The optimum temperature and pH for the growth of KOR-2 were 38°C and 6.8 to 7.0, respectively, while the optimum NaCl concentration essential for KOR-2 growth was 1.0% (w/v). KOR-2 tolerated ampicillin, penicillin G, kanamycin, spectromycin, and tetracycline. In contrast, the cell growth was inhibited by chloramphenicol. Phylogenetic analysis of 16S rRNA and *mcrA* genes revealed the relatedness between KOR-2 and *Methanoculleus bourgensis*.

**Conclusion:**

Based on the physiological and phylogenetic characteristics, KOR-2 was thought to be a new strain within the genus *Methanoculleus* and named *Methanoculleus bourgensis* KOR-2.

## INTRODUCTION

In the past decade, ruminal methanogens have attracted much research interest to mitigate ruminal methane (CH_4_) emission, as rumen CH_4_ emission accounts for about 17% of the global CH_4_ emission [1]. In addition, about 2% to 12% of the ingested feed energy is lost in the form of CH_4_ [2]. Studies related to ruminal methanogens are directed to understand their diversity and community structure, relationship with other ruminal microbes and feed efficiency, CH_4_ emission, and responses to dietary interventions.

The rumen provides a unique environment characterized by a relatively rapid passage rate and readily available carbon dioxide (CO_2_) and hydrogen (H_2_). This environment, therefore, facilitates the assembly of a community of archaea and is different from other anoxic habitats. Methanogens are the most dominant archaea in the rumen, and most of them are hydrogenotrophic rather than acetoclastic in spite of the high concentrations of acetates in the rumen [3]. H_2_ and CO_2_ produced from the fermentative pathways of other ruminal microbes are scavenged by rumen methanogens that also utilize formic acid and methyl-amines as substrates [4]. The interspecies H_2_ transfer in the rumen ecosystem prevents H_2_ accumulation and feedback inhibition. Most methanogens live freely in the rumen fluid or as members of the biofilm adhering to feed particles, whereas small portions of the ruminal methanogens are symbionts and may be either ectosymbionts or endosymbionts [5].

Given that methanogens are difficult to study through culture-based methods, many researchers have instead used culture-independent techniques such as real-time polymerase chain reaction (qPCR), denaturing gradient gel electrophoresis, and sequencing approaches, all of which have been valuable tools for the study of the biodiversity of complex microbial communities such as those in the rumen [6,7]. The diversity in the rumen methanogens is much smaller than that of rumen bacteria, and methanogens account for only 6.8% of total ruminal small subunit ribosomal ribonucleic acid (SSU rRNA) [8]. Archaea represent <3.3% of the total rRNA (both 16S and 18S) in the rumen [3]. The 16S rRNA gene sequences from cultured methanogens only account for approximately 0.7% of the total archaeal sequences of rumen origin, and several taxa have no single cultured representative [3]. In comparison with other anaerobic habitats from where methanogens have been isolated and classified into 28 genera and more than 100 species [9], the diversity and species richness of ruminal methanogens are quite low, reflecting the highly selective ruminal environment for methanogens [10]. To date, only 10 species of ruminal methanogens have been isolated as pure cultures [11,12]. *Methanobacterium formicicum*, *Methanobacterium bryantii*, *Methanobrevibacter ruminantium*, *Methanobrevibacter millerae*, *Methanobrevibacter olleyae*, *Methanomicrobium mobile*, *Methanoculleus olentangyi* (a later heterotypic synonym of *M. bourgensis*), *Methanosarcina barkery*, *Methanobrevibacter boviskoreani*, *Methanobacterium beijingense*, *M. marisnigri*, and *Methanosarcina mazei* (based on the RDP database). Here, we have isolated and studied the characteristics of a strain of *M. bourgensis* from the rumen of Holstein steers in Korea.

## MATERIALS AND METHODS

This study was approved by the Institutional Animal Care and Use Committee at the Chung-Ang University, Seoul, Korea (No. 2013-0047).

### Source of inoculum

Representative rumen contents were obtained from three ruminally cannulated Holstein steers (793±8 kg) 2 h after morning feeding. Holstein steers were offered typified commercial concentrates (Table 1) and rice straw at a ratio of 40:60. The steers had a free access to the diet and water. Fresh rumen contents (900 mL) were collected into bottles previously kept warm and filled with O_2_ free-CO_2_ gas and then filtered through four layers of gauze. The filtered rumen fluid was used as an inoculum to isolate methanogens. The concentrates were oven-dried at 60°C for 3 days, milled to pass through a 1-mm sieve, and analyzed for chemical composition using the appropriate AOAC [13] and Van Soest method [14].

### Medium

Methanogens are extremely sensitive to oxygen and need strict anoxic conditions; therefore, pre-reduced media are essential for their growth and isolation [6,7]. The methods used for the preparation of media and substrate solutions and culture techniques were those described by Hungate [15], as modified by Balch et al [16]. For enrichment culture and isolation, the medium was prepared based on the modification by Sowers and Schreier [17] and contained the following compounds (per liter distilled water): KH_2_PO_4_, 0.51 g; K_2_HPO_4_, 0.24 g; MgSO_4_·7H_2_O, 0.204 g; (NH_4_)_2_SO_4_, 0.255 g; NaCl, 1.02 g; CaCl_2_·2H_2_O, 0.134 g; vitamin solution, 10.0 mL; yeast extract, 1.0 g; sodium acetate, 50 mM; methanol, 50 mM; sodium formate, 50 mM; rumen fluid, 300 mL; NaHCO_3_, 5.0 g; resazurin, 0.001 g; 0.2 M cysteine hydrochloride, 15 mL; and 0.2 M Na_2_S·9H_2_O, 4.0 mL. Clarified rumen fluid was prepared by filtering the rumen fluid collected from the rumen of Holstein steers, followed by centrifugation at 13,000×*g* for 30 min. The rumen fluids were autoclaved at 121°C for 20 min and recentrifuged to obtain a clear yellow solution. The yellow solution (5%, v/v) was used to supply unknown growth factors. The medium was prepared under a H_2_/CO_2_ (80:20, v/v) gas phase at 173 kPa (25 psi) and its pH was adjusted between 6.8 and 7.0. The medium was sterilized by autoclaving at 121°C for 30 min.

### Enrichment and isolation

Enrichments were performed in the medium with pH 7.0 adjusted under H_2_/CO_2_ (80:20, v/v) gas phase. A 5-mL inoculum was added to vials containing medium (45 mL). To inhibit the growth of bacteria, streptomycin sulfate (7×10^4^ IU/L) and benzyl penicillin (2×10^10^ IU/L) were added to the medium. The inoculated medium was incubated at 38°C in the dark for 2 weeks. After detection of high levels of methane in the culture, 5 mL of the culture was anaerobically transferred into a new vial of sterile medium. Roll tubes containing the medium with 1.8% agar were prepared, followed by four successive transfers. Well-isolated colonies were withdrawn with Pasteur pipettes and transferred to culture tubes containing the medium under anaerobic condition. The culture tubes were sealed with butyl-rubber stoppers and repressurized with sterile filtered H_2_/CO_2_ (80:20, v/v) at 173 kPa (25 psi). The organism was re-isolated with the solid medium from the liquid cultures and inoculated on the bacterial growth medium to check for the purity of methanogen cultures. The bacterial growth medium contained peptone (2.5 g/L), yeast extract (2.5 g/L), d-glucose (0.5 g/L), d-cellobiose (0.5 g/L), and d-xylose (0.5 g/L). An antibiotic mixture containing four antibiotics (benzyl penicillin, 0.5 mg/mL; streptomycin sulfate, 0.5 mg/mL; vancomycin-HCl, 0.2 mg/mL; and ampicillin, 0.2 mg/mL) was prepared for the further purification of the isolated strain KOR-2. After purification, the isolate was incubated in the media without antibiotics.

### Physiological studies

Optimum growth temperature, pH, and NaCl concentration as well as substrate utilization and antibiotic tolerance were investigated to determine the physiological characteristics of the KOR-2 strain. Growth was confirmed by observing optical density (OD) at 660 nm (OD_660_) with a spectrophotometer (V-530, Jasco, Tokyo, Japan) and measuring the methane concentration in the gas phase using a gas chromatograph (GC-2010A, Shimadzu, Kyoto, Japan). All experiments for physiological studies were repeated twice.

The medium without acetate, formate, and methanol was prepared under N_2_ and used for substrate utilization studies. Anaerobic stocks of the filter-sterilized substrates (sodium formate, sodium acetate, trimethylamine, methanol, 2-propanol, and isobutanol) were prepared and separately added at a final concentration of 50 mM. Freshly grown cultures of the isolate were inoculated at 10% (v/v) and vials were incubated at 38°C for 20 days. The medium under H_2_/CO_2_ served as the control.

The optimum growth temperature in the medium was determined at optimum pH. Vials inoculated with 10% (v/v) culture were incubated at temperatures ranging from 20°C to 50°C. The vials were pressurized every other day with H_2_/CO_2_ to ensure an adequate supply of substrate.

The optimum growth pH in the medium was determined at the optimum temperature, with pH values ranging from 4.0 to 9.0. Media with pH above 4.0 were prepared by adding sterile sodium carbonate (Na_2_CO_3_) to media at pH 4.0 until the required pH value was reached. Medium with pH 4.0 was produced by removing sodium bicarbonate (NaHCO_3_) from the medium and cooling it under a CO_2_ headspace.

The sensitivity of KOR-2 strain to ampicillin, penicillin G, spectromycin, kanamycin, tetracycline, and chloramphenicol (all at a concentration of 100 μg/mL) was tested. Aliquots (5 mL) of the cultures were inoculated into fresh media containing one of the six antibiotics. KOR-2 strain was incubated for 1 week at 38°C. The tolerance to antibiotics was determined by comparing the growth of cultures containing these antibiotics to that of the control.

The salinity range of the isolate was tested at NaCl concentrations ranging from 0.5% to 3.0% at an interval of 0.5%. Media with various concentrations of NaCl were prepared by adding a sterile anoxic stock solution of 58.44 g/L NaCl.

### Microscopy

An Olympus BX41 phase-contrast microscope (Olympus, Tokyo, Japan) was routinely used to observe cells. Motility was determined by the hanging-drop method using a glass cavity slide.

### DNA extraction and G+C content

Culture samples of KOR-2 strain grown in the media were used for DNA isolation (FastDNA SPIN kit for soil, MP Biomedicals, Irvine, CA, USA) following the manufacturer’s instructions. DNA integrity was evaluated on 1% agarose gels and DNA concentration was determined using a Nanodrop (ND 2000, Thermo Fisher Scientific, Waltham, MA, USA). The DNA G+C content was analyzed from thermal denaturation profiles [18]. The experiments for DNA extraction and G+C content of KOR-2 strain were conducted several times until clear results were obtained.

### Polymerase chain reaction amplification of 16S rRNA genes

The primer pair Ar109f (5′-ACKGCTCAGTAACACGT-3′) [19] and Ar1383r (5′-CGGTGTGTGCAAGGAGCA-3′) [20] was used to obtain 16S rRNA gene amplicons (~1,350 bp). Reaction mixtures contained the following components in a final volume of 20 μL within a 200-μL PCR tube: 2 μL of PCR buffer (Takara, Tokyo, Japan), 2 μL of dNTP mix (35 mM), 0.5 μL of each primer (10 pmol/μL), 0.1 μL of Taq DNA polymerase (Takara, Japan), 1.0 μL of template DNA sample (100 ng), and 13.9 μL of molecular grade water (Severn Biotech Ltd, Kidderminster, Worcester, UK). PCR was started by immediately placing the reaction tubes into a preheated (94°C) thermal cycler (PCR Thermal Cycler Dice, Takara, Japan). The thermal program was as follows: Initial denaturation step (94°C, 4 min) followed by 30 cycles of denaturation (94°C, 30 s), annealing (55°C, 30 s), and extension (72°C, 90 s). After a final extension step (72°C, 6 min), samples were stored at 4°C until further analysis. Each PCR run included a positive control of DNA extracted from pure cultures and a negative PCR control where molecular grade water was substituted for the DNA template. The DNA product was analyzed using gel electrophoresis (1.2% w/v agarose gel stained and run at 100 V for 30 min in 1× tris-acetate ethylene-diamine-tetraacetic acid [EDTA; TAE] buffer with 5 μL aliquots of each DNA product). TAE buffer comprised 40 mM Tris base, 20 mM acetic acid, and 0.5 M EDTA (pH 8.0). The gel was run with 2.5 μL of Ladder I DNA quantification marker (TNT research, Seoul, Korea) and imaged using the Bio Imaging System (Daihan, Seoul, Korea). Photographs were obtained using Wise Capture II software (Daihan, Korea).

### Polymerase chain reaction amplification of *mcrA* genes

The *mcrA* gene fragments were amplified using the primer combinations MLf (5′-GGTGGTGTMGGATTC ACACARTA YGCWACAGC-3′) and MLr (5′-TTCATTGCRTAGTTWGG RTAGTT-3′), yielding ~490-bp amplicons [21]; ME1 (5′-GCM ATGCARATHGGWATGTC-3′) and ME2 (5′-TCATKGCRTA GTTDGGRTAGT-3′), yielding ~740-bp amplicons [22]; and MR1 (5′-GACCTCCACTWCGT VAACAACGC-3′) and ME2, yielding amplicons of ~1,100 bp [23]. Denaturation, annealing, and extension were carried out at 96°C (15 s), 55°C (30 s), and 72°C (90 s), respectively, with MLf/MLr primers, and 94°C (40 s), 50°C (45 s), and 72°C (90 s), respectively, with ME1/ME2 and MR1/ME2 primers.

### Phylogenetic and sequencing analyses

Purification of PCR products was performed with the AccuPrep PCR purification kit (Bioneer, Daejeon, Korea). PCR products were sequenced using the BigDye terminator cycle sequencing kit on ABI 3730XL capillary DNA Sequencer (Applied Biosystems, Thermo Fisher Scientific Inc., Carlsbad, CA, USA). 16S rRNA and *mcrA* gene sequences from the isolated strain were compared to the similar sequences obtained from GenBank using the BLAST program. Phylogenetic analysis was conducted using MEGA 4.0 [24]. We examined eight additional 16S rRNA sequences (*M. bourgensis* MS2 [HE964772], *M. palmolei* [Y16382], *M. receptaculi* ZC-2 [DQ787476], *M. taiwanensis* CYW4 [KM111599], *Methanofollis tationis* [AF 095272], *Methanoregula formicica* SMSP [CP003167], *Methanogenium marinum* AK-1 [DQ177344], and *Methanoplanus limicola* DSM 2279 [CM001436]). Phylogeny was further confirmed with *mcrA* gene sequences (*M. bourgenisis* MS2 [AB AB300787], *M. bourgensis* RC/ER [AB300785], *M. bourgensis* CB1 [AB300786], *M. bourgensis* MAB1 [KJ708788], *M. bourgensis* MAB2 [KJ708789], *M. chikugoensis* NBRC 101202 [AB 703634], *M. chikugensis* [AB300779], *M. thermophius* DSM 2624 [AF313804], and *M. thermophiles* [AB300783]).

### Nucleotide sequence accession number

The 16S rRNA and *mcrA* gene sequences of strain KOR-2 determined in this study were deposited in the GenBank database under No. JQ973736 and KF773774.

## RESULTS AND DISCUSSION

### Characterization of the isolated methanogen

A new methanogen was isolated from the rumen of Holstein steers. The methanogenic enrichment culture was obtained after repeated transfer in the presence of antibiotic mixtures for 2 months. Visible colonies on solid media appeared after 2 weeks of incubation at 38°C. Surface colonies were about 0.5 to 1.0 mm in diameter, yellow, circular, and convex. Only one strain, designated as KOR-2, was characterized in detail.

**Figure 1:**
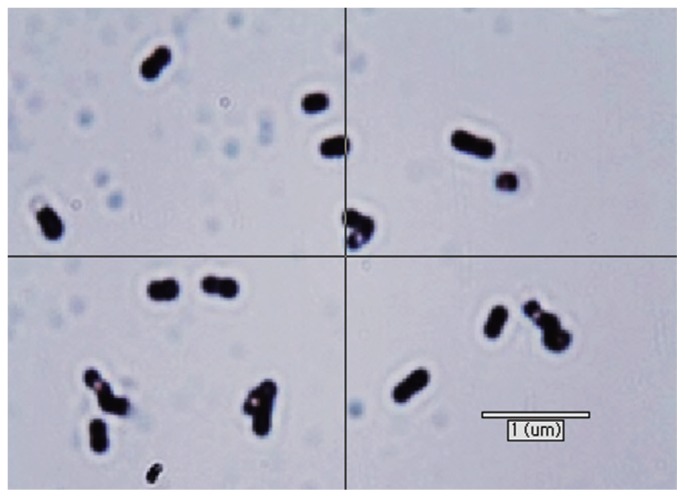
Phase-contrast microscopy of strain KOR-2. Bar indicates 1 μm.

Table 2 shows the comparison between the phenotypic and growth characteristics of KOR-2 strain and *M. bourgensis* MS2 [25–27]. KOR-2 cells were irregular cocci, non-motile, 0.2 to 0.5 μm in diameter, and occurred singly or in pairs (Figure 1). The growth of KOR-2 strain was observed at a temperature range of 25°C to 45°C, with the fastest growth reported at 38°C. The pH range suitable for its growth was 4.0 to 9.0, and the optimum pH for growth was 6.8 to 7.0. KOR-2 could grow well in salinity up to 3.0% (w/v) and the optimum NaCl concentration for the strain was 1.0%. This range of salinity is typical for halotolerant organisms. The G+C content of genomic DNA of KOR-2 strain was 55.5 mol%. KOR-2 used H_2_/CO_2_ and sodium formate (50 mM) but was unable to metabolize sodium acetate, methanol, trimethylamine, 2-propanol, and isobutanol as substrates for growth and methane production. The isolate was phenotypically similar to the compared strain *M. bourgensis*. Maestrojuán et al [26] reported that the cells of *Methanoculleus* are irregular cocci, 0.5 to 2.0 μm in diameter, and gram-negative and occur singly or in pairs. Some species are motile. Members of the family *Methanomicrobiaceae* have been found in a wide variety of anaerobic environments where methane is produced, such as the rumen of ruminant animals, anaerobic marine sediments, wetlands, and oil wells [28]. The genus *Methanoculleus* contains nine species [29]. *M. bourgensis* (basinym: Methanogenium bourgense), a species including the former M. olentangyi comb. nov. isolated from a tannery by-product enrichment culture inoculated with sewage sludge [25] (basinym: *Methanogenium olentangyi*) [30], and *M. oldenburgensis* [31] were described as junior heterotypic synonyms. *M. hydrogenitrophicus* was obtained from a wetland soil. *Methanoculleus* strains, including *M. bourgensis* MS2, are obligate anaerobes that produce methane from H_2_/CO_2_ or formate [25,29]. Some species also produce methane from secondary alcohols and CO_2_. Acetate is generally required as a carbon source, and additional growth factors may be required. Two types of methanogens, *Methanosarcina* sp. and *Methanosaeta* sp., were known to be capable of metabolizing acetate [32]. KOR-2 has typical mesophilic temperature optima, whereas other *Methanoculleus* species have higher or lower optima. Furthermore, some species are moderately thermophilic [33]. The G+C content of the DNA varies between 55.5 and 62.9 mol%. The G+C content value for KOR-2 was within the range for the strains belonging to the genus *Methanoculleus*, as reported by Ollivier et al [25]. Based on the morphology and substrate utilization, KOR-2 strain may exhibit the basic characteristics of *M. bourgensis*.

KOR-2 was able to grow in the presence of ampicillin, penicillin G, kanamycin, spectromycin, and tetracycline in the media, while the cell growth was inhibited by chloramphenicol. Sensitivity to antibiotics was reported for a limited number of species of the family *Methanomicrobiaceae*, *M. receptaculi* [34], *Methanofollis aquaemaris* [35], M*ethanofollis formosanus* [36], and *Methanogenium frittonii* (a later heterotypic synonym of *M. thermophilus*) [37] and members of the genus *Methanoplanus* [38]. Cells are sensitive to chloramphenicol and resistant to penicillin, ampicillin, kanamycin, vancomycin, and streptomycin [29]. *Methanogenium frittonii*, *Methanofollis aquaemaris*, and *Methanofollis formosanus* were sensitive to tetracycline, but *Methanoplanus* spp. were reported to be resistant [29]. *M. receptaculi*, but not *Methanogenium frittonii*, was inhibited by erythromycin [29]. Hilpert et al [39] found that archaea were insensitive to many antibiotics that inhibit eubacteria and eukaryotes, such as those inhibiting the synthesis or cross-linkage of the peptide subunit of murein or those suppressing RNA synthesis. Thus, KOR-2 was insensitive to antibiotics used in this study except for chloramphenicol. Chloramphenicol as a protein inhibitor interferes with the cell membrane function of methanogens. However, it is unknown if this insensitivity to chloramphenicol was associated with the impermeability of the cytoplasmic membrane or inactivation of the antibiotic by the cell, rather than the absence of a particular target for the antibiotic [39].

### Molecular characterization

**Figure 2:**
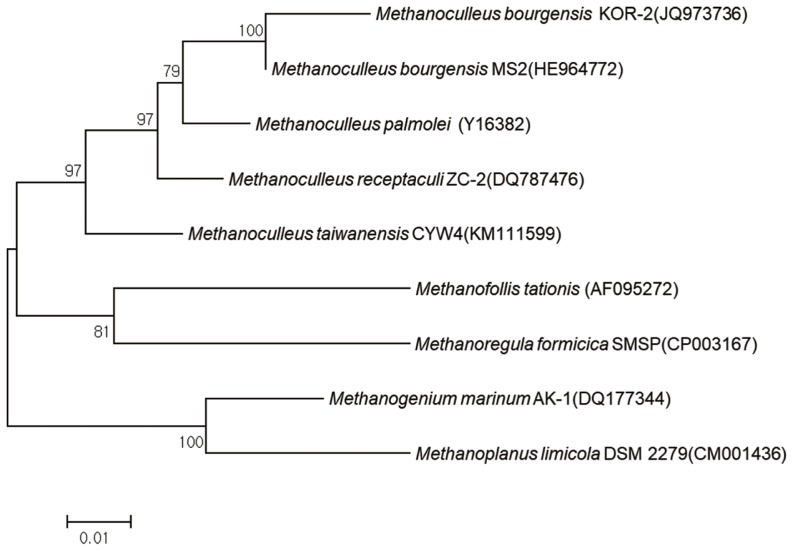
Phylogenetic dendrogram of 16S rRNA gene sequences showing the position of the *Methanoculleus bourgensis* strain KOR-2 relative to other species of the genus *Methanoculleus* as well as selected reference sequences of methanogens. *Methanofollis tationis* and *Methanoregula formicica* were used as outgroup references. The evolutionary distances were computed using the maximum composite likelihood method [24]. Bootstrap values are shown at nodes (percentages of 500 replicates). GenBank accession numbers are indicated. A bar represents 0.01 substitutions per nucleotide position.

**Figure 3:**
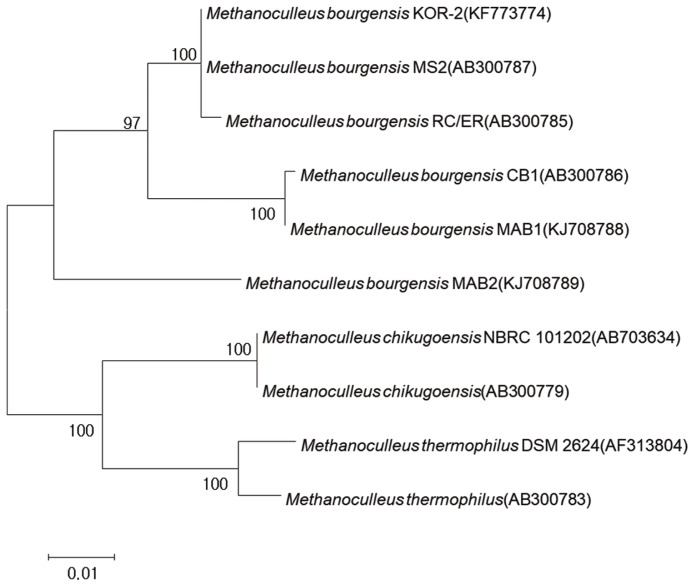
Phylogenetic tree of deduced *mcrA* gene sequences indicating the relationship between the *Methanoculleus bourgensis* strain KOR-2 and members of the genus *Methanoculleus*. GenBank accession numbers are indicated. Bootstrap values are shown at nodes (percentages of 500 replicates). A bar represents 0.01 substitutions per nucleotide position.

Polymerase chain reaction of 16S rRNA yielded an amplicon size of 1,350 bp. *mcrA* gene-based amplification was also used for identification purposes and yielded a product size of 790 bp. Comparative 16S rRNA gene sequence analysis showed that the strain was affiliated with the order *Methanomicrobiales*. The closest relatives of KOR-2 strain were *M. bourgensis* MS2 (98%, sequence similarity), *M. palmolei* (97%), *M. receptaculi* ZC-2 (96%), *Methanofollis tationis* (91%), and *Methanoplanus limicola* DSM 2279 (90%) (Figure 2). KOR-2 showed a small difference in physiological characteristics (Table 2) and 98% sequence similarity to the 16S rRNA gene of *M. bourgensis* MS2 (Figure 2). The RDP Release 11 (Update 3) reported that a total of 8623 sequences of archaeal 16S rRNA gene sequences were originated from the rumen of ruminants. About 90% of these sequences were assigned to methanogens. These sequences were classified to 10 known genera, with *Methanobrevibacter* being represented by 63.2% of all the sequences followed by *Methanosphaera* (9.8%), *Methanomicrobium* (7.7%), and *Methanobacterium* (1.2%) [3]. Among the gene sequences of the rumen, the 5 sequences of *Methanoculleus* were identified, in which 4 sequences were recovered from isolates including one gene from *M. bourgensis* KOR-2. It is noted that the genus of *Methanoculleus* is not probably major species in the rumen. The *mcrA* gene sequence also indicated that KOR-2 strain was a member of the order *Methanomicrobiales*. The closest relatives of KOR-2 strain based on the *mcrA* gene sequence were *M. bourgensis* MS2 (100%) and *M. chikugoensis* (94%) (Figure 3). All phylogenetic results were similar in the experiment. Luton et al [40] stated that the *mcrA* gene sequence may be alternatively used instead of the 16S rRNA-based sequence methods, demonstrating far greater diversity than that observed with 16S rRNA gene sequences in the methanogen population.

On the basis of morphology, physiological characteristics, and phylogenetic analyses described above, the strain was identified as a new strain of *M. bourgensis* and named as KOR-2.

## Figures and Tables

**Table 1:** Composition of the concentrate mix

Items	
Ingredients	g/kg of dry matter
Ground corn	30.2
Wheat	21.0
Soybean meal	24.0
Rice bran	10.0
Tapioca	178.0
Sesame oil meal	14.0
Palm kernel meal	410.8
DDGS	220.0
Molasses	50.0
Condensed molasses soluble	10.0
Salt	3.0
Limestone	20.0
CaCO_3_	7.0
Minerals and vitamin mixture1)	7.0
Chemical composition	
Dry matter	882.7
Crude protein	145.0
Ether extract	67.7
Crude fiber	126.8
Undegradable protein	73.6
Ash	74.9
NFE	471.0
NFC	198.3
ADF	230.8
NDF	396.9
TDN	710.2

DDGS, dried distillers grain with solubles; NFE, nitrogen-free extract; NFC, non-fiber carbohydrate; ADF, acid detergent fiber; NDF, neutral detergent fiber; TDN, total digestible nutrients.

1) Minerals and vitamin mixture: vitamin A, 28,000 IU; vitamin D_3_, 4,000 IU; vitamin E, 80 IU; Mn, 80 ppm; Zn, 100 ppm; Fe, 70 ppm; Cu, 50 ppm; Co, 0.5 ppm; I, 2.0 ppm; Se, 1.0 ppm.

**Table 2:** Comparative characteristics of KOR-2 strain isolated from the rumen of Holstein steers1)

Characteristics	KOR-2	MS2T
Cell morphology	Coccoid	Coccoid
Cell width (μm)	0.2–0.5	1.0–2.0
Temperature for growth (°C)		
Range	25–45	30–49
Optimum	38	37
pH for growth		
Range	4.0–9.0	5.5–8.0
Optimum	6.8–7.0	6.7
NaCl for growth range (%)		
Range	0.5–3.0	0–4.0
Optimum	1.0	1.0
DNA G+C content (mol%)2)	55.5 (Tm)	59 (Bd)
Substrate utilization		
H_2_/CO_2_	+	+
Formate	+	+
Acetate	−	−
Methanol	−	−
Trimethylamine	−	−
2-propanol	−	−
Isobutanol	−	−
Tolerance for antibiotics		
Ampicillin	+	ND
Penicillin G	+	ND
Spectromycin	+	ND
Kanamycin	+	ND
Tetracycline	+	ND
Chloramphenicol	−	ND

−, negative; +, positive; ND, no data available.

1) Data for strain KOR-2 are from this study, and those for strain MS2T are retrieved from Ollivier et al [25] and Maestrojuán et al [26].

2) Determined by buoyant density analysis (Bd) and melting point analysis (T_m_).
